# Prevalence of peripheral arterial disease and its association with claudication in individuals with type 2 Diabetes Mellitus: a prospective cross-sectional study in Brazil

**DOI:** 10.1590/1677-5449.202400852

**Published:** 2025-04-18

**Authors:** Gislaine Bonete da Cruz, Cibele Teresinha Dias Ribeiro, Camila Maciel de Oliveira, Rosangela Roginski Rea, Fernando Augusto Lavezzo Dias

**Affiliations:** 1 Universidade Federal do Paraná – UFPR, Curitiba, PR, Brasil.; 2 Universidade de São Paulo – USP, Faculdade de Medicina – FM, São Paulo, SP, Brasil.

**Keywords:** peripheral arterial disease, Diabetes Mellitus, diabetes complications, prevalence, ankle-brachial index, doença arterial periférica, Diabetes Mellitus, complicações do diabetes, prevalência, índice tornozelo-braço

## Abstract

**Background:**

Few studies have described the prevalence of peripheral arterial disease (PAD) in the diabetic population of Brazil.

**Objectives:**

To evaluate the prevalence of PAD and its association with the presence of claudication and to compare risk factors for atherosclerosis between subjects with and without PAD.

**Methods:**

An analytical, observational, prospective, cross-sectional study was conducted with 150 volunteers with type 2 Diabetes Mellitus (T2DM) treated at a university endocrinology outpatient clinic (Federal University of Paraná, Curitiba, Brazil) and assessed using the ankle-brachial index (ABI) and the Edinburgh Claudication Questionnaire.

**Results:**

ABI was evaluated in 143 volunteers (7 did not meet the inclusion criteria). Six individuals had an ABI > 1.4. Excluding these cases and adopting an ABI ≤ 0.9 to identify PAD, the prevalence of PAD was 14.6% (20 of 137), 15.8% in men (6 of 38), and 14.1% in women (14 of 99). We identified twenty-five participants with borderline ABI (18.2%). Claudication was present in 25% of subjects with PAD; however, only 15% had typical claudication. A significant association with the presence of typical claudication was observed only when ABI values were stratified above or below 1.0 (p = 0.04, Fisher’s exact test). Participants with PAD were older (median difference: 5.5 years, 95% CI 3.0 to 9.0, p < 0.001) and tended to have a longer duration of DM and higher BMI.

**Conclusions:**

The prevalence of PAD in T2DM volunteers with no prior screening was 14.6% and 75% were asymptomatic.

## RESUMO

**Contexto:**

Há poucos estudos que descrevem a prevalência da doença arterial periférica (DAP) na população diabética no Brasil.

**Objetivos:**

Avaliar a prevalência de DAP e sua associação com a presença de claudicação e comparar fatores de risco para aterosclerose entre indivíduos com e sem DAP.

**Métodos:**

Estudo analítico, observacional, prospectivo e transversal, realizado com 150 voluntários com Diabetes Mellitus tipo 2 (DM2) em tratamento no ambulatório universitário de endocrinologia da Universidade Federal do Paraná (Curitiba, Brasil). Os participantes foram avaliados pelo índice tornozelo-braquial (ITB) e pelo Questionário de Claudicação de Edinburgh.

**Resultados:**

O ITB foi avaliado em 143 voluntários (7 não atenderam aos critérios de inclusão). Seis indivíduos apresentaram ITB > 1,4. Excluindo esses casos e adotando o ITB ≤ 0,9 para a definição de DAP, a prevalência da doença foi de 14,6% (20 de 137), sendo 15,8% em homens (6 de 38) e 14,1% em mulheres (14 em 99). Além disso, 25 participantes apresentaram ITB limítrofe (18,2%). A claudicação esteve presente em 25% dos indivíduos com DAP, mas apenas 15% apresentaram claudicação típica. A associação entre DAP e claudicação típica foi significativa apenas quando os grupos foram estratificados para valores de ITB acima ou abaixo que 1,0 (p = 0,04, teste exato de Fisher). Os participantes com DAP eram mais velhos (diferença de medianas: 5,5 anos; intervalo de confiança de 95% de 3,0-9,0; p < 0,001) e apresentaram tendência a maior duração do DM e maior índice de massa corporal.

**Conclusões:**

A prevalência de DAP em indivíduos com DM2, sem triagem prévia, foi de 14,6%, sendo 75% assintomáticos.

## INTRODUCTION

Peripheral Arterial Disease (PAD) is a growing public health challenge due to its increasing prevalence worldwide. In 2019, it was estimated to affect 1.52% of the global population, corresponding to approximately 113 million cases.^1^ Prevalence is higher in women and increases with age, exceeding 9% in individuals over 70 years of age (age-specific prevalence peaks at 70–74 years) and reaching approximately 20% in those aged 90-94 years.^1^

PAD is associated with elevated risk of mortality, specifically cardiovascular morbidity and mortality.^2^ The adjusted relative risk for incident PAD associated with Diabetes Mellitus (DM) is 1.96 (95% CI 1.29–2.63) in women and 1.84 (95% CI 1.29–2.86) in men.^3^ A recent article analyzing data from the Global Burden of PAD Study^1^ found that high fasting plasma glucose has become the secondary critical risk factor (others are high blood pressure and smoking) predicted to cause greatest PAD disease burden, especially in lower sociodemographic index regions.^4^ In Brazil, there are few studies describing the prevalence of PAD in the diabetic population.^5-9^

The ankle-brachial index (ABI) (≤ 0.90) has high specificity and accuracy, ranging from 83.3% to 99.0% and 72.1% to 89.2%, respectively, and was able to reliably identify patients with serious ≥ 50% stenosis, as shown by Xu et al. (2010)^10^ in a systematic review. Sensitivity varies among studies, with early studies reporting sensitivity of up to 94-97%,^11^ while others have since reported very low percentages.^10^ This discrepancy is probably due to the lack of consistent confirmation of PAD and diversity of study populations.^11^ Even though sensitivity and specificity may vary among DM patients, the test is nevertheless recommended for preliminary diagnosis for PAD due to its simplicity and convenience.^12^ Alternatives to ABI are the toe-brachial index (TBI), recommended to be performed when resting ABI is inconclusive, i.e. >1.40; segmental leg pressures with pulse volume records and/or Doppler waveforms, to help delineate the anatomic level of PAD (recommended for chronic symptomatic PAD); and sometimes angiography, when further delineation of arterial anatomy and site of obstruction will impact plans for therapy.^12^

Therefore, our aims were to assess the prevalence of PAD, screening volunteers for the first time using the ABI test, in a population of people with diabetes in the Metropolitan Area of the City of Curitiba (PR), Brazil, and to compare the risk factors for atherosclerosis between those who have and those who do not have PAD. We also aimed to assess the presence of claudication and its association with normal and abnormal ABI values.

## METHODS

### Participants and study design

An analytical, observational, prospective, cross-sectional study was conducted to identify the prevalence of Peripheral Artery Disease and its association with claudication symptoms in a sample of the Brazilian population affected by Diabetes Mellitus. We followed the STrengthening the Reporting of OBservational studies in Epidemiology (STROBE) recommendations to report the study. All volunteers were being monitored and treated for type 2 DM at a university endocrinology outpatients clinic (Endocrinology Service – SEMPR, Hospital das Clínicas of the Federal University of Paraná, Curitiba, Brazil) and were invited to participate in the study (convenience sampling), based on the inclusion criteria described below, at their routine consultations (during the period from October 2016 to March 2019). The estimated population served by the service annually was 2,160 subjects.

The sample size was initially estimated using prior data published by Sales et al., in 2015,^5^ and Becks et al., in 1995,^13^ assuming a prevalence of approximately 17%, absolute precision of 4%, and level of significance of 5%, resulting in 280 subjects. Owing to time and resource restrictions, 150 volunteers were recruited during the study period.

None of the volunteers had previously been screened for PAD (using ABI or any other method) and consented to be included in the study, which involved a broader investigation into cardiovascular autonomic control and sexual function (data not shown). Therefore, we present data from the assessment of circulatory status in this population. All procedures were approved by the institution’s Research Ethics Committee (Ethical approval # 1.759.971, CAAE 09506319.2.0000.0096).

We included adult subjects (over the age of 30 years) regardless of the time of initial diagnosis, if they did not present with amputation, active ulcers in the ankle and foot region, or any other condition that makes it impossible to measure blood pressure, such as fistula, lymphedema, or revascularization of the lower or upper limbs.

To assess risk factors related to atherosclerosis (smoking, dyslipidemia, hypertension, and body mass index) we accessed clinical data and biochemical analysis results available in the subjects’ medical records from the previous 12 months.

### Assessment of PAD using ABI

PAD was assessed by a trained person using the ABI test following the methods and classification cutoffs described by the American Heart Association.^2,12^ In brief, after resting for 10 minutes, blood pressure was measured twice in the supine position, in the upper limbs (brachial arteries) and ankles (both dorsalis pedis and posterior tibial), using a sphygmomanometer (model Durashock DS44, WelchAllyn/Tycos®, Germany) with a suitable cuff for the limb circumference, with the aid of a portable Doppler ultrasound machine (model DV-10, Microem®, 10MHz). A third measurement was taken if there was a greater than 10% difference between the first two measurements. The ankle-brachial index was determined for each leg by dividing the higher systolic pressure value in the ipsilateral dorsalis pedis and posterior tibial arteries by the higher systolic pressure value in the left or right brachial artery.

The reference values used were according to the guidelines from 2016 (the same as in the recently updated 2024 guidelines)^2,12^ and reported as abnormal (ABI ≤0.90), borderline (ABI 0.91-0.99), normal (ABI 1.00-1.40), or non-compressible (ABI >1.40). We followed the most recent recommendation to report resting ABI using stratification that includes borderline ABI.^12^ Borderline ABI has been associated with higher risk of mortality and PAD,^14^ and with prevalence of micro and macrovascular complications in DM2.^15^ We therefore considered this when analyzing associations with claudication.

### Assessment of presence of claudication

We assessed the presence of intermittent claudication using the validated Portuguese language version of the Edinburgh Claudication Questionnaire.^16^ Positive claudication was defined as responses to questions 1 = “yes” AND 2 = “no” AND 3= “yes” AND 5 = “usually disappears in 10 minutes or less” AND 6 = marked “calf” and/or “thigh” and/or “gluteus region”. Claudication was defined as negative for any other combination of responses. Subjects positive for claudication were defined as typical claudicants (also defined as definite claudicants in the original questionnaire) if they indicated pain in the calf, regardless of whether pain at other sites was also marked. Atypical claudication is classified as pain reported in the thigh or buttocks in the absence of any calf pain. We evaluated whether this categorization would have improved the questionnaire’s sensitivity and specificity for identification of individuals with an abnormal ABI, as described below.

To determine the association between ABI and claudication assessed using the Edinburgh questionnaire, we stratified the groups using subject’ lowest ABI value to allocate them to the following groups: ABI from 0.91-1.4 and ABI ≤ 0.9, and also classified them using the stratification considering borderline ABI, i.e. groups with ABI from 1.0 -1.4 (normal values) and ABI < 1.0 (borderline and values compatible with PAD) before performing the statistical test described below to assess the predictive value of the Edinburgh questionnaire for identification of PAD.

### Statistical analysis

For statistical analysis, we assessed the normality of the data using the Shapiro-Wilk test. We used Student’s *t* test for parametric data and the Mann-Whitney test for non-parametric data to compare the means (or medians) between subjects with PAD and without PAD identified by ABI (i.e., ≤ 0.90), while the chi-square test was used to compare percentages. We used contingency tables and Fisher’s exact test to assess the sensitivity, specificity, and positive predictive value of the Edinburgh questionnaire to detect PAD. Diagnostic accuracy was calculated by dividing the proportion of individuals correctly classified by the questionnaire (sum of true positives and true negatives) by the total number of individuals evaluated.

## RESULTS

**Figure 1:**
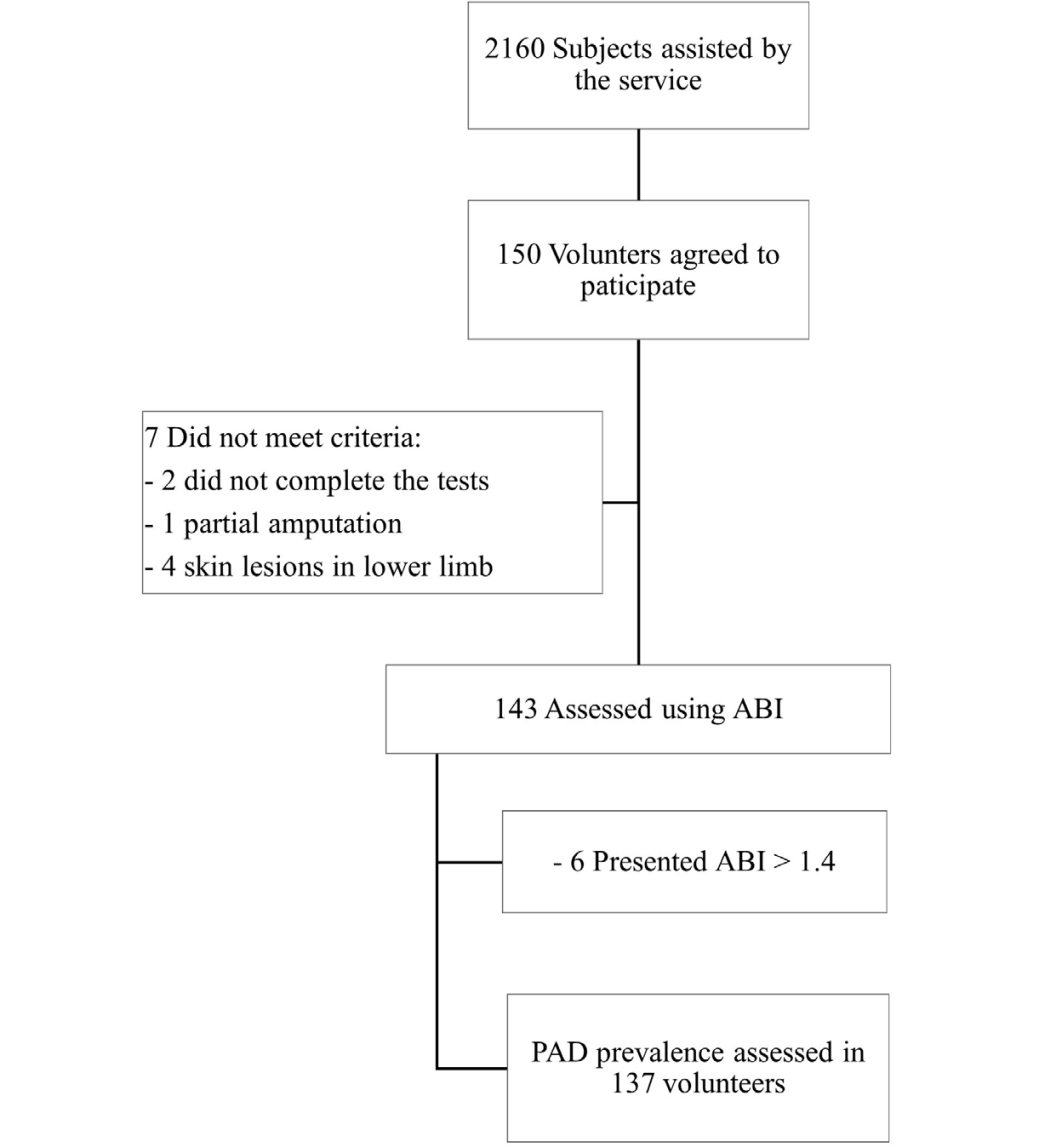
Flow chart of the study population.

A total of 150 volunteers agreed to participate in the study, seven did not meet the inclusion criteria, and ABI was assessed in 143 volunteers (Figure 1). Six participants had ABI > 1.4. The characteristics of the study population are summarized in Table 1.

**Table 1:** Characteristics of the study population.

	**n**	**Median (interquartile range) or %**
Right ABI	142/143	1.09 (0.99-1.16)
Left ABI	141/143	1.10 (1.01-1.16)
Lower ABI	143/143	1.05 (0.97-1.13)
Age (years)	143/143	61 (57-65)
BMI (Kg/m^2^)	142/143	30.1 (27.4-33.8)
DM duration (years)	138/143	14.0 (10.0-20.0)
HbA1c (%)	137/143	8.1 (7.0-9.4)
Total Cholesterol (mg/dL)	130/143	165.0 (137.8-193.0)
HDL (mg/dL)	135/143	44.0 (36.0-52.0)
Triglycerides (mg/dL)	132/143	128.5 (87.3-180.3)
LDL (mg/dL)	133/143	90.0 (72.0-114.5)
Insulin treatment (%)	143/143	71.33
Hypertension treatment (%)	143/143	83.92
Dyslipidemia treatment (%)	143/143	79.02
Current smokers (%)	143/143	12.59

Data are presented as median and interquartile range of percentage of occurrence. We considered the most recent clinical data for biochemical analyses available in the subject’s medical record, not exceeding 12 months. Missing data may be due to noncompressible arteries. ABI – Ankle Brachial Index; BMI – Body Mass Index; HbA1c - Glycated hemoglobin; HDL - High-density lipoprotein, LDL - Low-density lipoprotein. Lower ABI refers to the lowest value for each volunteer, regardless of laterality.

Abnormal ABI (≤0.9 or >1.4) was present in 18.2% of the sample (26 out of 143). Excluding inconclusive ABI (i.e., > 1.4) and adopting ABI ≤ 0.9 as the cut-off to identify participants with ABI compatible with PAD, the prevalence of PAD was 14.6% (20 out of 137), 15.8% in men (6 out of 38) and 14.1% in women (14 out of 99). We identified twenty-five participants with borderline ABI (18.2%).

As summarized in Table 2, compared to those with ABI above 0.91, participants with ABI ≤ 0.9 were older and tended to have had DM for a longer time. Comparing subjects with normal ABI (i.e. ABI > 1.0) to subjects with PAD (index ≤ 0.9), the differences persisted for ABI and age and so did the trends to differences in DM duration (median difference: 6.50, 95% CI 0.00 to 9.00, p = 0.053) and BMI (median difference: 1.65, 95% CI -0.100 to 5.20, p = 0.056). We did not find any differences between the groups in classical risk factors for atherosclerosis, such as the percentages with hypertension or dyslipidemia or of smokers.

**Table 2:** Comparison of clinical and anthropometric data between people with Diabetes Mellitus and ABI results above or below 0.9.

	**n**	**ABI 0.91-1.40 (n=117)**	**ABI ≤0.90 (n=20)**	**p**
Right ABI	137/137	1.10±0.10	0.80±0.13	<0.001
Left ABI	137/137	1.11±0.09	0.84±0.16	<0.001
Lower ABI	137/137	1.06 (1.00-1.130)	0.82 (0.66-0.88)	<0.001
Age (years)	137/137	60.0 (56.0-64.0)	65.5 (61.2-69.0)	<0.001
BMI (Kg/m^2^)	136/137	30.10 (27.3-33.2)	31.4 (29.4-36.5)	0.13
DM duration (years)	132/137	13.5 (10.0-20.0)	19.5 (11.0-27.5)	0.07
HbA1c (%)	131/137	8.1 (6.9-9.2)	7.7 (7.0-10.2)	0.80
Total Cholesterol (mg/dL)	124/137	167 (137-196)	150 (145-190)	0.78
HDL (mg/dL)	129/137	44.0 (36.0-51.0)	45.5 (35.2-55.5)	0.60
Triglycerides (mg/dL)	126/137	130 (86-182)	126 (101-175)	0.65
LDL (mg/dL)	117/132	95.0 (72.5-115.0)	82.0 (74.7-104.0)	0.61
Insulin treatment (%)	137/137	70.1	80.0	0.37
Hypertension treatment (%)	137/137	82.0	95.0	0.14
Dyslipidemia treatment (%)	137/137	77.8	85.0	0.46
Current smokers (%)	137/137	10.3	20.0	0.21

Data are presented as mean ± standard deviation for parametric data and as median (interquartile range) for non-parametric data. We used Student’s *t* test for parametric data, the Mann-Whitney test for non-parametric data, and the chi-square test for comparison of percentages. We considered the most recent clinical data for biochemical analyses available in the subject’s medical record, not exceeding 12 months. ABI – Ankle Brachial Index; BMI – Body Mass Index; HbA1c - Glycated hemoglobin; HDL - High-density lipoprotein, LDL - Low-density lipoprotein. Lower ABI refers to the lowest value for each volunteer, regardless of laterality.

In the subset of participants who had PAD, 25% (5 out of 20) reported claudication symptoms and 15% (3 out of 20) were classified as typical claudicants. Table 3 presents data from the Edinburgh Claudication Questionnaire. We assessed the associations between presence or absence of typical claudication and ABI levels to evaluate the predictive value of the Edinburgh questionnaire for detecting PAD. There was no association between presence of typical claudication and groups of volunteers classified by ABI as normal, including borderline values, i.e., ABI from 0.91 – 1.40 compared to abnormal values, i.e., ABI ≤ 0.9. However, there was an association with presence or absence of typical claudication (p = 0.04 in Fisher’s exact test) when we allocated subjects to a group with normal ABI (1.0 to 1.4) or a group with ABI below 1.0 (which includes both borderline and abnormal ABI). In this case, the positive predictive value of typical claudication for predicting PAD was 0.6364 (95% CI 0.3538 to 0.8483) (sensitivity = 0.156, 95% CI 0.077 to 0.288; specificity = 0.956, 95% CI 0.894 to 0.983), with accuracy of approximately 69%.

**Table 3:** Data from the Edinburgh Claudication Questionnaire.

**ABI**	**n**	**Presence of Claudication (Typical or Atypical)**	**Presence of Typical Claudication**
1.00 - 1.40	92	13	4
0.91 – 0.99	25	8	4
≤ 0.9	20	5	3

ABI – Ankle Brachial Index.

## DISCUSSION

In the present study, we assessed ABI in 143 volunteers with DM2 and excluded subjects with non-compressible arteries (ABI > 1.4), finding that the prevalence of PAD was 14.6% (20 out of 137) with a similar distribution between men and women. Subjects with PAD were older, tended to have a higher BMI and DM duration, and were mostly asymptomatic (75%). There was only a significant association between typical claudication and ABI when comparisons were made between subjects with ABI values above or below 1.0.

The prevalence of PAD in subjects with DM found in our study using ABI is similar to the 13.7% PAD prevalence reported by Sales et al.^5^ in a prospective cross-sectional study that included a population with the same characteristics, followed at a university endocrinology outpatient clinic, and living in the northeastern region of Brazil (Natal, RN). In a retrospective study, Moreira et al.^8^ identified a 15.3% prevalence of PAD among patients with type 2 DM in the city of Viçosa, MG, Brazil. Felício et al.^9^ reported a higher prevalence of PAD (28%) in newly-diagnosed type 2 DM patients referred to tertiary care. Interestingly, the percentage found in the present study is also very similar to a recent much larger study (over 2000 subjects over 18 years of age) conducted in Sri Lanka, where the PAD prevalence in type 2 DM was 15.3%.^17^ PAD prevalence seems to be higher in the diabetic population than in the general population, since Makdisse et al.^6^ reported a prevalence of 10.5% in a general sample from different regions of Brazil.

The findings of the present study corroborate those of previous reports, including in the Brazilian population,^7–9^ demonstrating that individuals with type 2 DM and PAD are older and tend to have longer DM duration and higher BMI.

Most subjects with PAD assessed in our study (75%) did not report claudication symptoms. This shows there is a significant percentage of patients with asymptomatic and subclinical PAD in this population who, once identified, could benefit from preventive measures and possibly treatment to avoid progression of the disease. Furthermore, identifying individuals with peripheral artery obstruction would raise awareness of obstruction at other sites, such as coronary, carotid, and cerebral arteries.^12,18^ In the present study, we used the Edinburgh Claudication Questionnaire to assess claudication. The association between presence of typical claudication and ABI values was only significant when subjects were stratified as having ABI from 1.0 -1.4 (normal) or ABI below 1.0 (i.e., grouping borderline and PAD diagnosed by ABI together). Therefore, the presence of typical claudication identified by the questionnaire may indicate that further investigation is needed for subjects with borderline ABI because they are at higher risk of developing PAD^14^ in the future and have a higher risk of mortality based on recent studies.^14,15^ We found higher specificity than sensitivity for the Edinburgh Claudication Questionnaire and accuracy of approximately 69%. Other studies have also found that the Edinburgh Claudication Questionnaire presents high specificity and low sensitivity, with comparable accuracy (around 53%).^19,20^

One limitation of the present study was the small number of volunteers recruited, which makes extrapolations of the results to the general Brazilian population difficult. It is also known that medial arterial calcinosis is more frequent in DM patients, reducing the diagnostic precision of ABI.^21,22^ In the present study, we did not perform additional tests such as toe-brachial index or assessment of ABI after exercise to assess the presence of PAD in individuals with non-compressible arteries or with borderline ABI. This could also be a limitation of the study. Additionally, the biochemical data were based on medical records and were not collected prospectively, which may contribute to inaccuracy in the group comparisons.

## CONCLUSION

We found a significant prevalence of PAD (14.6%) identified using ABI in individuals with type 2 DM in a sample of the Brazilian population who had not previous been screened using ABI or any other diagnostic method, the majority of whom (75%) were asymptomatic.
